# Position of the *dentifera*-group in the *Coronatella*-branch and its relocation to a new genus: *Magnospina* gen. n. (Crustacea, Chydoridae, Aloninae)

**DOI:** 10.3897/zookeys.586.8209

**Published:** 2016-05-04

**Authors:** Francisco Diogo R. Sousa, Lourdes Maria Abdu Elmoor-Loureiro, Sandro Santos

**Affiliations:** 1 Núcleo de Estudos em Biodiversidade Aquática, Programa de Pós-graduação em Biodiversidade Animal, Universidade Federal de Santa Maria – UFSM, Av. Roraima 1000, Camobi, CEP 97105-900, Santa Maria, RS, Brazil; 2 Laboratório de Biodiversidade Aquática, Universidade Católica de Brasília – UCB, QS7 lote 1, Bloco M, sala 204, CEP 71966-700, Taguatinga, DF, Brazil

**Keywords:** Alona broaensis, Alona dentifera, Alona siamensis, Celsinotum, Leberis, male, morphology, Ovalona

## Abstract

*Magnospina* **gen. n.** was created to relocate species of the *dentifera*-group from *Alona* sensu lato (Crustacea: Cladocera) and include *Magnospina dentifera* **comb. n.** and *Magnospina siamensis* **comb. n.** The synapomorphies of the *Magnospina* **gen. n.** are (1) basal spines longer than 2/3 of the postabdominal claw, (2) presence of 1–4 large denticles, broad at their bases, protruding downwards, without setules between them. Morphological traits such as habitus, rostrum and postabdomen shape, armature of IDL setae, number of setae on the exopod of limb III are also important in the distinction between *Magnospina* **gen. n.** and other genera from the *Coronatella*-branch. The morphology of *Magnospina dentifera* **comb. n.** male confirms the closer relationship with the clade composed by the *elgans*-group from *Alona* sensu lato, *Ovalona* and *Leberis*, but the external morphology, morphology of the postabdominal claw, basal spine and setae 2–3 of IDL support their separation from any of the group cited. It is concluded that the *Coronatella*-lineage of Aloninae is composed of the genera *Coronatella*, *Anthalona*, *Karualona*, *Bergamina*, *Extremalona*, *Ovalona*, *Celsinotum*, *Leberis* and *Magnospina* **gen. n.** The *elegans*-group from *Alona* sensu lato also belongs to *Coronatella*-lineage, but still need formal allocation.

## Introduction

The taxonomic status of *Alona dentifera* (Sars, 1901) (Crustacea: Cladocera) was discussed by Sinev et al. (2004). In this study, the authors relocated *Alonella dentifera* to the genus *Alona* Baird, 1843 based on the absence of typical morphological traits of the subfamily Chydorinae Dybowsky & Grochowski, 1894 *emend.* Frey, 1967 and presence of some morphological traits of Aloninae Dybowsky & Grochowski, 1894 *emend.* Frey, 1967. Because of the polyphyletic nature of *Alona* (Sacherová and Hebert 2003, Elmoor-Loureiro 2004, Eliáz-Gutiérrez et al. 2008; Van Damme et al. 2010), allocations of species groups to different genera were made: *Phreatalona*, which corresponds to the *protzi*-complex (Van Damme et al. 2009); *Coronatella*, which corresponds to the *rectangula*-complex (Van Damme and Dumont 2008a); *Brancelia* (Van Damme and Sinev 2011), which corresponds to the *hercegovinae*-complex; and *Anthalona* Van Damme, Sinev & Dumont, 2011, which includes species of the *verrucosa*-complex (Van Damme et al. 2011a). Most recently, Sinev (2015a) included the *pulchella*-group in *Ovalona* Van Damme & Dumont, 2008.

Likewise, the position of *Alona dentifera* is doubtful because its morphology is very different from that of the “true *Alona*”, which is represented by the *quadrangularis*-group only (Van Damme and Dumont 2008b; Van Damme et al. 2010). Van Damme and Dumont (2008a) suggested that *Alona dentifera* belongs to a large lineage of *Alona* sensu lato, named the *Coronatella*-branch, and that it may be close to *Leberis*, as evidenced by molecular tools (Elias-Gutiérrez et al. 2008) or to the *Coronatella* genus. Although Chatterjee et al. (2013) consider *Alona dentifera* as a member of the *Coronatella* genus, this species seems to be part of a group with separate evolution, together with *Alona siamensis* Sinev & Sanoamuang, 2007. *Alona dentifera* and *Alona siamensis* share synapomorphies, as showed by Sinev and Sanoamuang (2007). Besides, the male morphology of *Alona dentifera* is quite different from *Coronatella* and *Leberis* (see description below).

Thus, our aim is to evaluate the morphological traits of *Alona dentifera*, based on original material from Brazil and Argentina, and to describe the adult male, for the first time. Additionally, we relocate *Alona dentifera* to a new genus, which also includes *Alona siamensis*.

## Methods

The description of the new genus was based on material collected in different localities in Brazil and Argentina (see material examined) and data from the literature (Sinev et al. 2004). The selected animals were transferred to drops of glycerol on slides and dissected under a stereomicroscope. The morphology of appendages and other structures was studied using a phase contrast microscope Olympus BX41. To enumerate the setae of limbs, we used the proposal of homology from Kotov (2000a, b), which presented stability when tested in different cladoceran groups (Kotov et al. 2010). Drawings were prepared using a camera lucida attached to a phase contrast microscope Olympus BX41.

The following abbreviations were used in the text, table and figures:

A1 antennule


A2 antenna


as accessory seta


CBS copulatory brush seta


en endite


ep epipod


ex exopod


fc filter comb


gfp gnathobasic filter plate


gn gnathobase


IDL inner distal lobe


il inner lobe


ms male seta


ODL outer distal lobe


P1-4 limbs I-IV


PA postabdomen


pep pre-epipod


s sensillum


### Depository abbreviations

FDRS
 Personal collection of Francisco Diogo R. Sousa 


CLLA
 Slides collection of the GEEA, at Universidade Católica de Brasília, Brazil 


ZMOU
Zoological Museum of Oslo University 


ZMMU
Zoological Museum of Moscow State University 


## Taxonomy

### Class Branchiopoda Latreille, 1817 Order Anomopoda Sars, 1865 Family Chydoridae Dybowsky & Grochowski, 1894 *emend.* Frey, 1967 Subfamily Aloninae Dybowsky & Grochowski, 1894 *emend.* Frey, 1967

#### Magnospina gen. n.

Taxon classificationAnimaliaDiplostracaChydoridae

http://zoobank.org/8BA31D3E-9088-4642-B489-EB9DB45FE9FA

##### Type species of the genus.

*Magnospina dentifera* comb. n. = *Alona dentifera* (Sars, 1901).

##### Etymology.

The name “*Magnospina*” is derived from two Latin words, *magna* = large, long and *spina* = spine. The generic name refers to the long basal spine on the postabdominal claw.

##### Description.

**Parthenogenetic female.** *Habitus* without dorsal keel, ovoid or with moderate lateral compression, length 0.32–0.48 mm, maximum height before the mid-length of body; body height/length about 1.3-.17. *Head* Eye and ocellus of subequal or different sizes. Rostrum short from a lateral view, wide from a frontal view, not pointed, rounded or truncated; head shield wide, with the distance between the mandibular articulations higher than length of its posterior portion, with or without ornamentation; head pores absent or presents, in last case three connected main head pores, lateral head pores minute. *Labral keel* wide, oval and naked, apex not elongated. *Carapace* ornamentation not evident, slightly punctuated or with narrow longitudinal lines; valves armed with 40–53 setae internally inserted at the ventral margin and differentiated in three groups, setae from the anterior group markedly longer than median and posterior groups; ventral margin with a distinctive rounded angle at 1/3 of the length of the margin. Anteroventral corner of valves rounded; posteroventral corner armed with 1–4 large denticles, broad at their bases, protruding downwards, without setules between them; posterodorsal corner poorly defined. Posterior margin almost straight, armed with inner setules on the carapace which are not arranged in groups. *Antennule* not exceeding the tip of the rostrum, about 2.5 times as long as it is wide; three or four rows of setules on the antennular body. Antennular sensory seta about 1/2 length of antennular body. Nine aesthetascs of different length present in a distal position not exceeding the length of antennular body. *Antenna* with formula of antennal setae 003/113, spines 101/001; first segment of endopod and exopod elongated, about two times longer than the others segments; weak setules or spicules on the segments. First exopod segment with a narrow, naked or plumose seta, with length similar or slightly longer than the branches. Spine on the first endopod segment longer than second endopod segment. Apical spines slightly longer than the apical segments or about two times shorter than the apical segment itself. Three plumose apical setae not differentiated in size among themselves. *Postabdomen* approxamately 1.3–2.5 times as long as wide, narrowing distally. Dorsal margin weakly convex or straight. Preanal angle clearly prominent; preanal, anal and postanal margins of different length; postanal margin about 1.5–1.8 longer than anal margin, armed with 9–13 marginal denticles, of which the most distal (1–4) might be individualized, proximal denticles organized in clusters; 8–10 lateral fascicles with setules relatively weak. *Postabdominal claw* inserted on the projection of postabdomen, 1.3–1.5 times longer than anal margin; spinules on the ventral margin may be present; pecten of spinules on the internal and external face of claw, median pecten with strong spinules; base of the claw armed with 1–5 long and strong spinules. *Basal spine* almost straight, remarkably long, longer than 2/3 of the postabdominal claw length, with or without spinules on the dorsal margin. *First Maxilla* with two setulated setae. *Limb I* with epipod oval, with a finger-like projection. ODL with bisegmented seta, serrated from middle portion towards the distal portion; accessory seta implanted near the base of the ODL. IDL (en 4) with two robust setae (2–3), seta (1) rudimentary or absent; IDL setae 2–3 thick, armed with thick basal denticles. Endite 3 with four setae, anterior seta (1) shorter or similar in length to posterior setae (a-b); setae (a-b) of similar or different length; a sensillum might be present on the endite. Endite 2 with three posterior long setae (d-f) which differ strongly in length among themselves; seta (d) shorter than the seta (e), setae (e-f) with thick spinules on the lateral face; a sensillum might be present on the endite. Endite 3 armed with three posterior setae (g-i); seta (i) plumose, about 1/2 of the setae (g-h). Ejector hooks relatively short. Ventral face of the limb with six-seven groups of setules organized in clusters. *Limb II* with exopod elongated, short seta present which might be plumose, about two-three times shorter than exopod itself. Inner portion armed with eight scrapers not specialized and decreasing in length towards distal portion, but with some denticles on the scrapers 6–8; anterior soft setae absent; gnathobase armed with four elements, filter comb armed with seven setae, of which two proximal are shorter than the others. *Limb III* with pre-epipod rounded and setulated, epipod oval with a short finger-like projection. Exopod with four distal and two lateral setae; fifth and sixth setae differentiated in length, third and fourth setae long; second seta about 1.4–1.7 times longer than first setae. Setae 3–6 clearly plumose. Distal endite armed with three setae and one sensillum, setae 1–2 scraper-like of different length; third seta curved and armed with many bilaterally implanted setules (3); four plumose posterior setae present. Basal endite with four soft anterior setae increasing in length towards the gnathobase, a sensillum might be present. Gnathobase with three elements, filtercomb with seven setae. *Limb IV* with pre-epipod rounded or rectangular and setulated, epipod oval with a long finger-like projection. Exopod with six marginal setae; first and second setae long, not plumose; third seta plumose, short, about two times shorter than the second seta; fourth seta long and plumose; fifth and sixth setae plumose and with similar lengths; Distal endite with four setae (1–4), one scraper-like (1), three flaming-torch-like (3–4); flaming-torch setae not modified. Basal endite with three slightly setulated soft setae. Gnathobase armed with a setulated seta shorter than the length endite itself, filter comb with five setae. *Limb V* with pre-epipod rounded or rectangular and setulated, epipod oval with a long finger-like projection. Exopod not divided in lobes, armed with four plumose setae. Setae 2–4 of subequal lengths; first seta about two-three times shorter than the other setae. Internal lobe wide, oval and with long setules apically and laterally implanted; two setulated setae on the inner face which are shorter than the length of lobe itself. Filter comb with one or without seta. *Limb VI* absent.

*Adult male*. As for *Magnospina dentifera* comb. n.

##### Diagnosis of the genus.

**Parthenogenetic female.** *Habitus* ovoid, without dorsal keel. *Head* with rostrum wide, not pointed; head shield wide with distance between mandibular articulations higher than length of its posterior portion, main head pores absent in adults of *Magnospina dentifera* comb. n. or with three connected main head pores in *Magnospina siamensis* comb. n.; lateral head pores absent (*Magnospina detifera* comb. n.) or present (*Magnospina siamensis* comb. n.). *Labral keel* wide and naked, apex not elongated. *Carapace* ornamentation not evident, punctuated or with narrow longitudinal lines; valves armed with 40–53 setae internally inserted at the ventral margin and differentiated in three groups, setae from the anterior group markedly longer than median and posterior groups; ventral margin with a distinctive rounded angle at 1/3 of the margin length; posteroventral corner armed with 1–4 large denticles, broad at their bases, protruding downwards, without setules between them. *Antennule* not exceeding the tip of the rostrum, nine aesthetascs of different lengths present distally. *Antenna* with formula of antennal setae 003/113; spines 101/001; basal segments on the exopod and endopod about two times longer than the other segments; weak setules or spicules on the segments. *Postabdomen* narrowing distally, preanal angle prominent; postanal margin armed with 9–13 marginal denticles which the most distal (1–4) might be individualized, proximal denticles organized in clusters; eight-10 lateral fascicles with weak setules. *Postabdominal claw* inserted on the projection of postabdomen, longer than anal margin; spinules on the ventral margin may be present; pecten of spinules on the internal and external face of the claw, base of claw armed with 1–5 long and spinules. *Basal spine* remarkably long, longer than 2/3 of postabdominal claw length, with or without spinules on the dorsal margin (absent in *Magnospina siamensis* comb. n.). *Limb I* with endite 1 armed with three setae (g-i); IDL (en 4) with two robust setae (2–3), seta 1 rudimentary (*Magnospina dentifera* comb. n.) or absent (*Magnospina siamensis* comb. n.); IDL setae 2–3 thick, armed with thick basal denticles. *Limb II* without soft setae; short seta on the exopod; scrapers not specialized, but with some denticles, especially on scrapers 6–8; gnathobase armed with four elements, filter comb armed with seven setae, of which two proximal are shorter than others. *Limb III* with six setae on the exopod, third and fourth setae long; distal endite armed with three setae and one sensillum; gnathobase with three elements, filter comb with seven setae. *Limb IV* relatively short, six setae on the exopod; third seta plumose, short, about two times shorter than the second seta; flaming-torch setae on the distal endite not modified; gnathobase armed with a setulated setae shorter than the length of endite itself, filter comb with five setae. *Limb V* relatively short, setae 3–4 of exopod subequal in length; filter comb reduced, with one short seta in *Magnospina dentifera* comb. n. and none in *Magnospina siamensis* comb. n.. *Limb VI* absent.

*Adult male. Habitus* smaller than female (Figure 27). *Postabdomen* strongly narrowing distally. *Postabdominal claw* short and ticker than female (Figures 30–31). *Basal spine* about half-length of postabdominal claw, with tip forked (Figure 31). *Limb I* with two setae on the IDL (en4), setae armed with denticles; male seta with tip slightly curved; copulatory hook with one projection on the tip (Figures 32–33).

##### Differential diagnosis.

**Figures 1–7.**
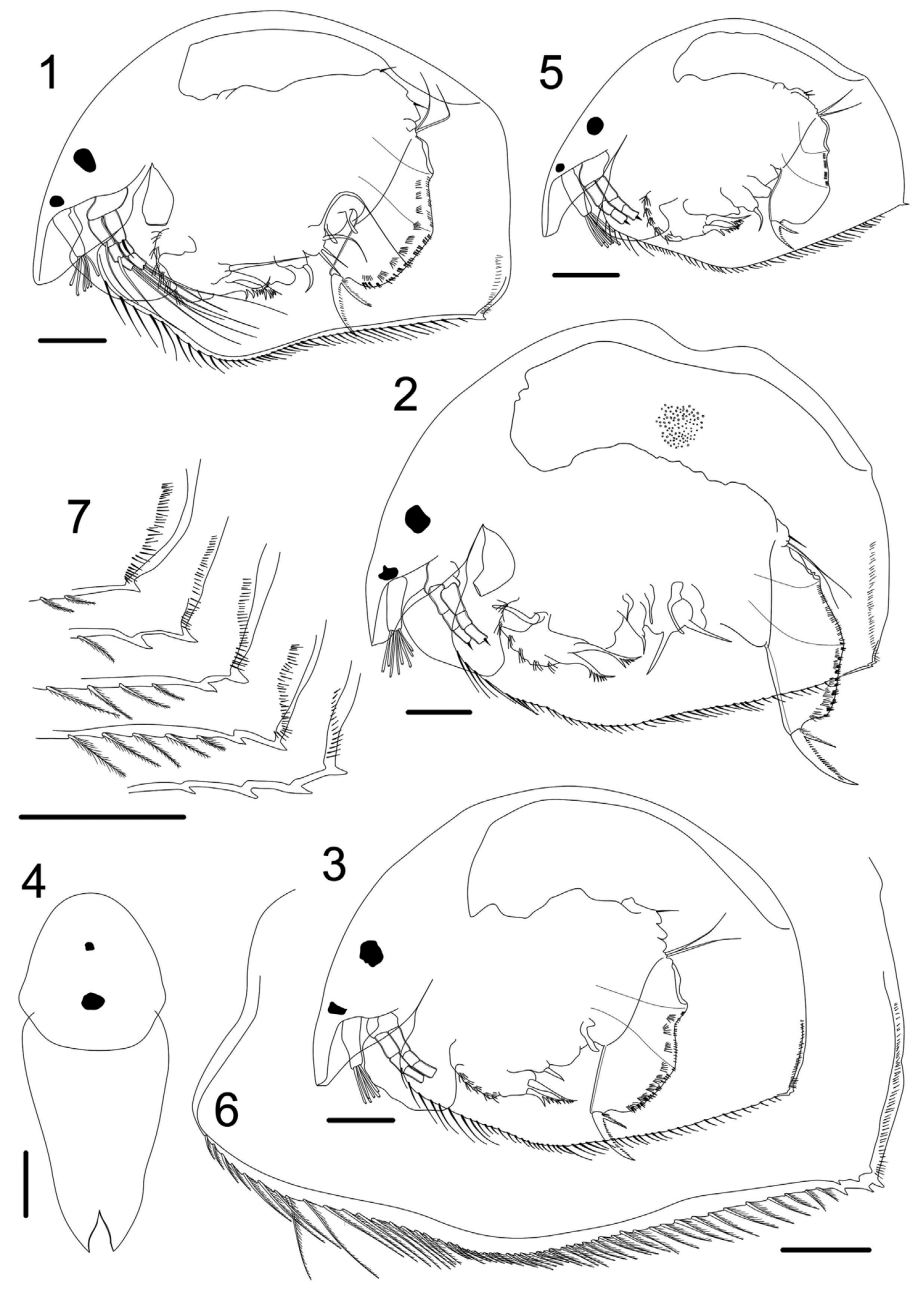
*Magnospina dentifera* comb. n., parthenogenetic female. **1** *habitus* from Pantanal, Mato Grosso do Sul, Brazil **2** *habitus* from São Paulo, Brazil **3–4** *habitus* from San Pedro, Argentina parthenogenetic female adult from **5** *habitus*, parthenogenetic female juvenile from San Pedro, Argentina **6** ventral margin of carapace from Distrito Federal, Brazil **7** denticles on the posteroventral margin of carapace. Scale bars: 50 µm.

The synapomorphies of *Magnospina* gen. n. are (1) basal spines longer than 2/3 of postabdominal claw, (2) presence of 1–4 large denticles, broad at their bases, protruding downwards, without setules between them. *Magnospina* gen. n. can also be differentiated from the genus *Coronatella* because it has a distinctive rounded angle at 1/3 of the length of the ventral margin, marginal setae of valves differentiated in three groups, setae from anterior group markedly longer, wide rostrum, postabdomen narrowing distally; the males of *Coronatella* do not bear two lateral aesthetascs on the antennules. *Magnospina* gen. n. differs from *Anthalona* in the presence of a distinctive rounded angle at 1/3 of the length of the ventral margin, sacks underneath lateral head pores (cosmaria) being absent, shape of postabdomen, poorly developed setules of the lateral fascicles, morphology of IDL (which does not have specialized denticles), and armature of limb I; the males of *Anthalona* also do not bear lateral aesthetascs on the antennules. The new genus differs from *Karualona* in the morphology of IDL setae, shape of the postabdomen, poorly developed setules of the lateral fascicles, seta on the exopod of limb II (present in *Magnospina* gen. n. and absent in *Karualona*) and endite basal of limb IV armed with three flaming-torch; the antennule of *Karualona* males bear just one lateral aesthetasc. *Magnospina* gen. n. is closer to *Leberis* according to Eliáz-Gutiérrez et al. (2008); however, it is distinguished by the presence of long setae on anterior group of ventral margin of the carapace, in the morphology of setae 2–3 of the IDL (Figures 18–19), presence of seta on exopod of limb II, absence of a dorsal keel (Figures 1–5), and presence of a long basal spine on postabdominal claw of postabdomen (Figures 13–16); males of *Leberis* also do not bear denticles on the posteroventral corner of valves. *Magnospina* gen. n. differs from *Celsinotum* Frey, 1991 in the absence of a dorsal keel, absence of spine-like setae on the posterior portion of valves, presence of relatively long apical (endopod and exopod) and basal (endopod) spines on the segment of the antenna, long basal spine on the postadbominal claw, absence of a rudimentary seta (i) on endite 1 of limb I (in *Magnospina* gen. n. setae (i) is developed). The new genus differs clearly from *Bergamina* Elmoor-Loureiro, Santos-Wisniewski & Rocha, 2013 in morphology of postabdomen, presence of denticles on the posteroventral margin of valves and absence of anterior seta between endites 1–2 of limb I (see Elmoor-Loureiro et al. 2013). *Magnospina* gen. n. differs from *Extremalona* Sinev & Shiel, 2012 in general morphology, presence of denticles on the posteroventral margin of valves, postabdomen morphology and armature of setae 2–3 of IDL; male of *Extremalona* also bears six lateral aesthetascs on the antennules. *Ovalona* Van Damme & Dumont, 2008 has a well-developed seta 1 on the IDL, endite 1 of the limb I without seta (i) and exopod of the limb III armed with seven setae. *Magnospina* gen. n. does not present any of aforementioned morphological traits to *Ovalona*. Table 1 shows the main differences and similarities between genera of the *Coronatella*-branch.

**Figures 8–15.**
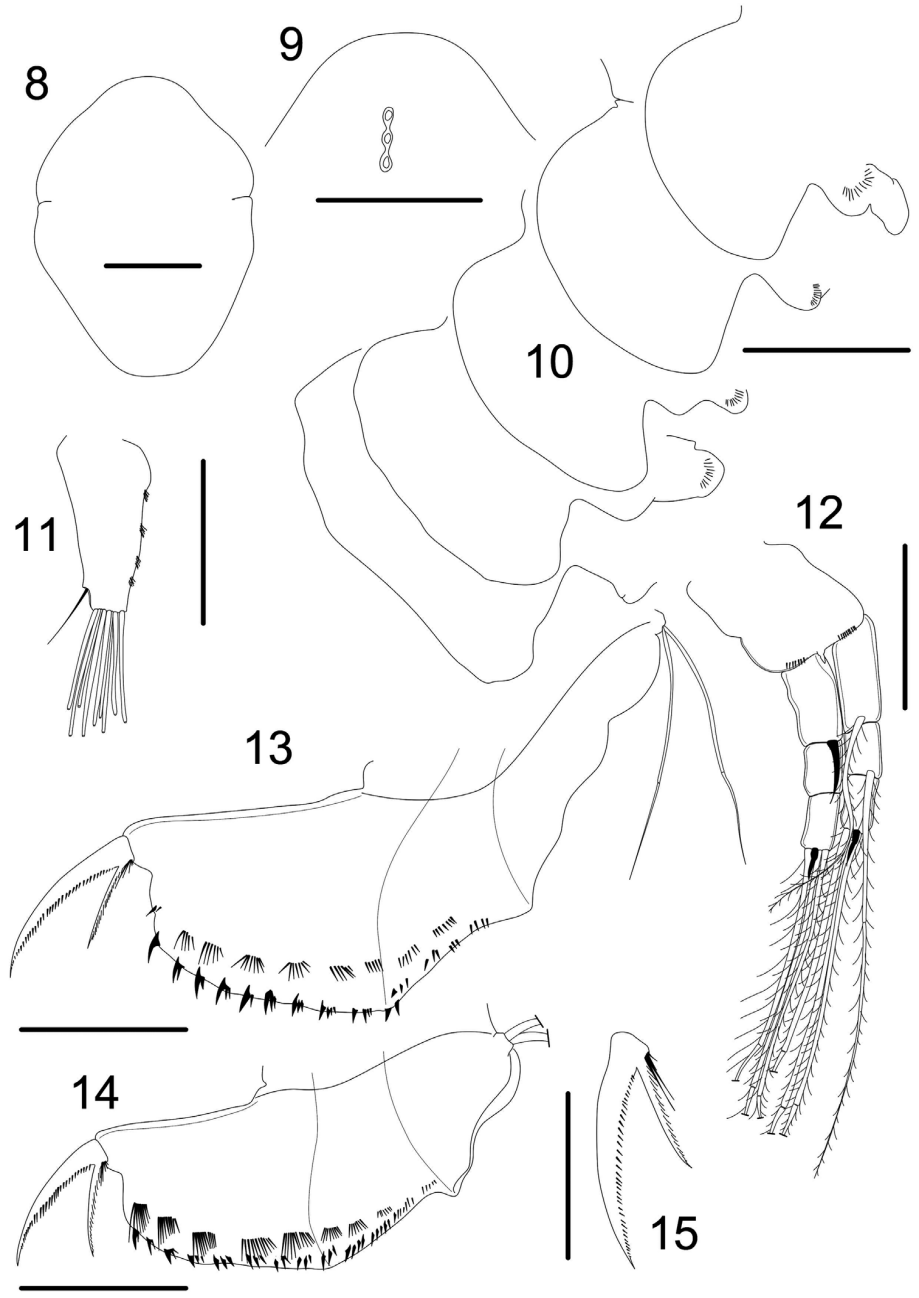
*Magnospina dentifera* comb. n., parthenogenetic females. **8** head shield **9** main head pores, female juvenile **10** labral kell **11** antennule **12** antenna **13 –14** postabdomen **15** postabdominal claw. Scale bars: 50 µm.

**Figures 16–26.**
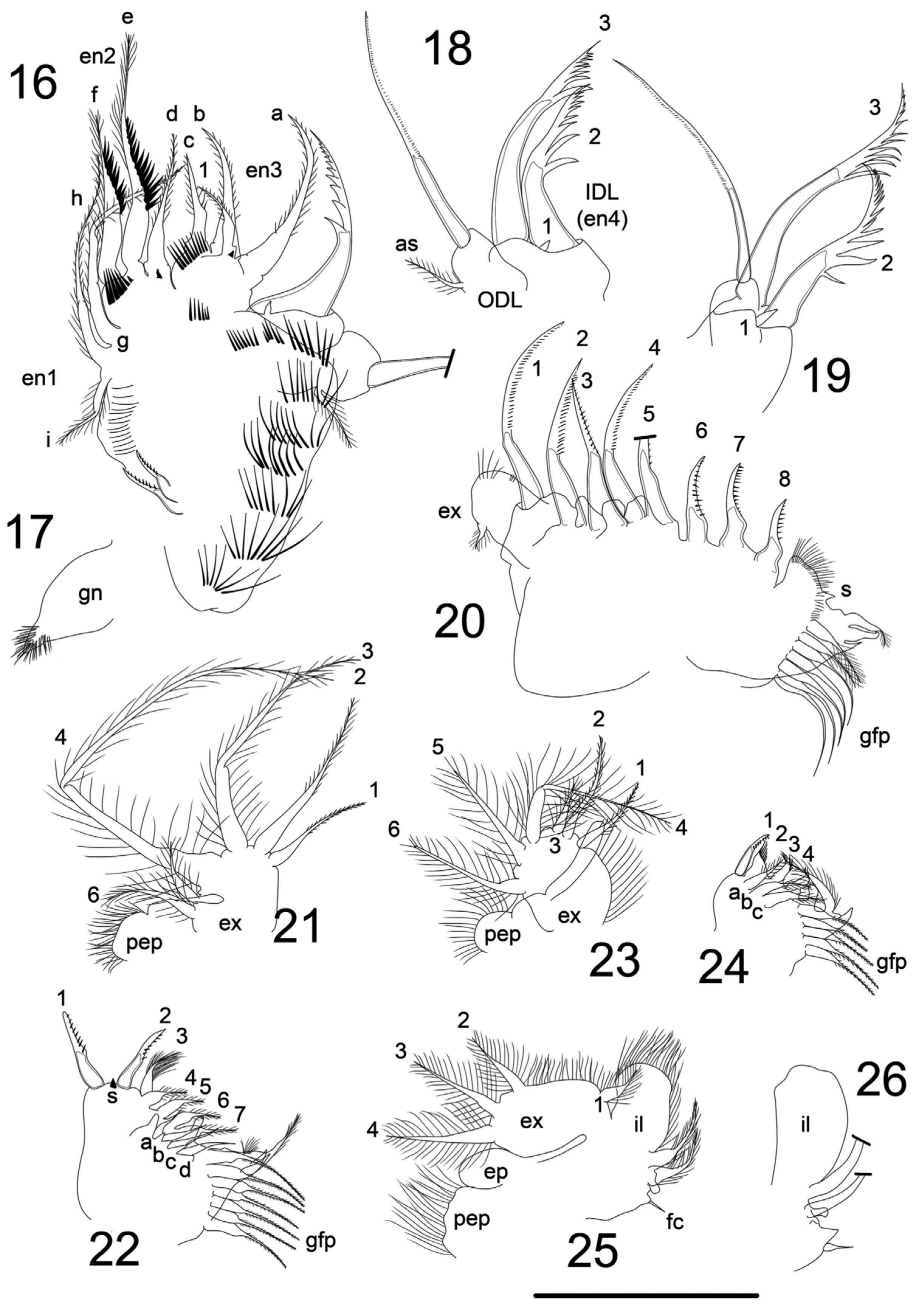
*Magnospina dentifera* comb. n., adult parthenogenetic females. **16** limb I **17** limb I, gnathobase **18–19** limb I, IDL and ODL **20** limb II **21** limb III, exopod **22** limb III, endites **23** limb IV, exopod **24** limb IV, endites **25** limb V **26** limb V, internal lobe. Scale bars: 50 µm.

**Figures 27–33.**
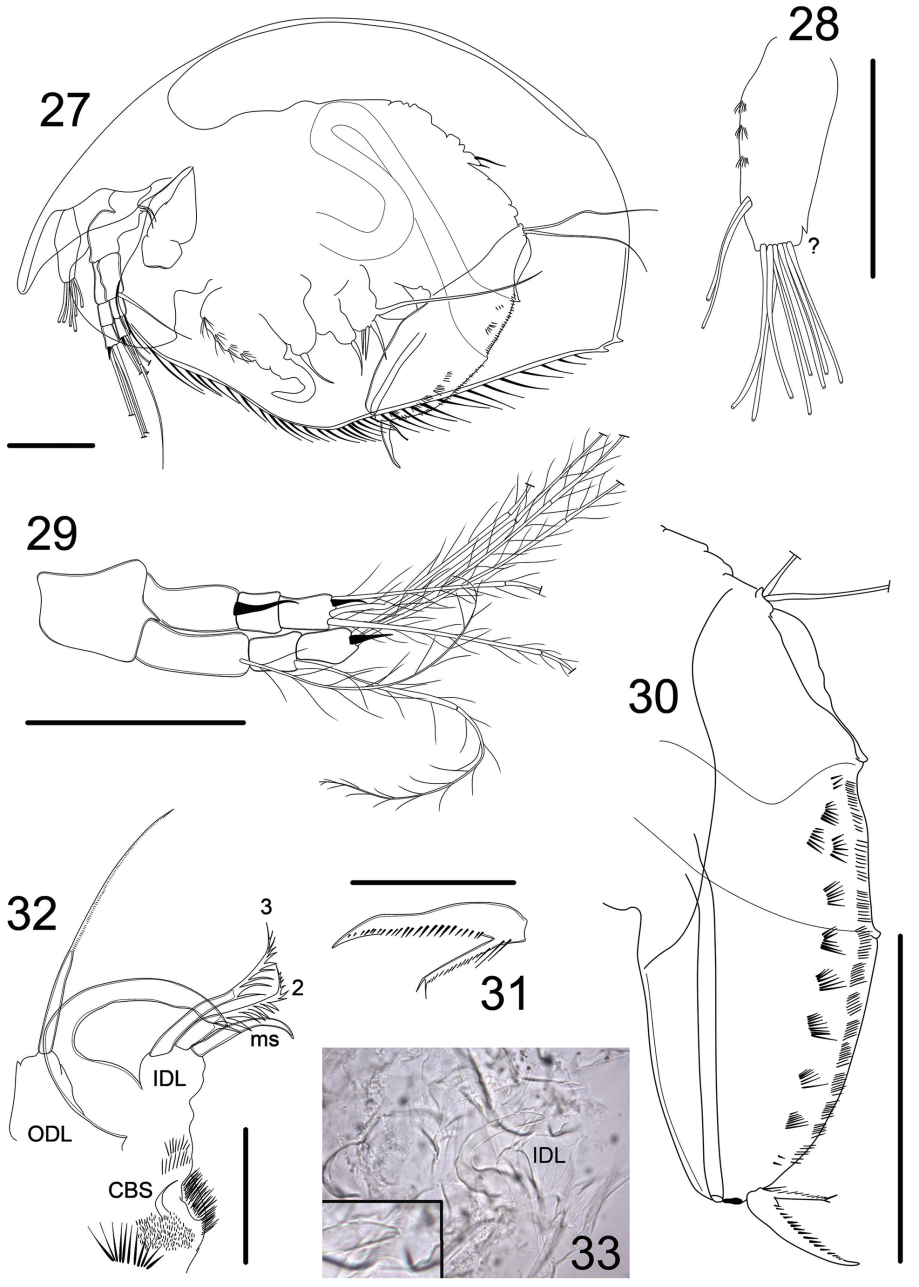
*Magnospina dentifera* comb. n., adult male from Henrique Pond, Brasília National Park, Distrito Federal, Brazil. **27** *habitus* **28** antennule **29** antenna **30** postabdomen **31** postabdominal claw **32** limb I, IDL and ODL **33** limb I, copulatory hook. Scale bars: 50 µm (**27–30)**; 25 µm (**31–33**).

**Table 1.** Main differences and similarities between genera related to *Coronatella*-branch. To date, there is no description of males of *Bergamina*. From *Karualona*, only the male of *Karualona iberica* is known (see Alonso and Pretus 1989). From *Magnospina* gen. n., only the male of *Magnospina dentifera* comb. n. is known.

	*Extremalona*	*Ovalona*	*Leberis*	*Celsinotum*	*Magnospina*	*Coronatella*	*Anthalona*	*Karualona*	*Bergamina*
**Female characters**									
Maximum length	0.5	0.35–0.7	0.5–1.1	0.53–0.9	0.32–0.48	0.25–0.6	0.3–04	0.4–0.45	0.68
Dorsal keel	absent	absent	present	present	absent	present in one species	absent	absent	absent
Labral denticle	absente	absent	absent	absent	absent	present in one species	absent in three species	absent	absent
Rostrum	rounded	rounded	truncated	truncated	rounded or truncated	rounded	rounded	rounded	rounded
Main head pores	three connected	three connected, connection interrupted or absent	three connected	three connected	absent or three connected	three connected	two connected	two connected	three connected
Lateral head pores	minute	minute, except in one species	minute	minute	absent or minute	minute	specialized, with underneath sacks	minute	minute
A2 – spine on the apical segments	large	large	small	small	large	large	large	large	small
Valves – ventral margin with a distinctive rounded angle	absent	absent	absent or slightly expressed	absent	present	absent	absent	absent	absent
Valves – length of ventral setae from anterior group/posterior group	not differentiated	anterior longer than posterior	not differentiated	anterior shorter than posterior	anterior very longer than posterior	not differentiated	anterior longer than posterior in three species	anterior longer than posterior	not differentiated
Valves – denticles	absent	1–2 denticles in one species	absent	absent	1–5 large denticles	1–2 short denticles in two species	absent	short denticles in most species	absent
PA – shape of postanal ventral margin	short, narrowing	short and rounded or elongated and narrow	elongated, narrowing	elongated, narrowing	elongated, narrowing	short, rounded	short, rounded	short, rounded	elongated, straight
PA – basal spine	short	short	short	well-developed, shorter than 2/3 of postabdominal claw	well-developed, longer 2/3 of postabdominal claw	short	short	reduced or short	short
PA – setae on the lateral fascicles	well- developed	well- developed	weakly developed	well- developed	weakly developed	well- developed	well- developed	well- developed	weakly developed
P1 – IDL seta 1	well- developed	well- developed	rudimentary	rudimentary	rudimentary or absent	rudimentary or absent	absent	well- developed	absent
P1 – IDL setae 2–3	with thin setules	with thin setules	with hard setules	with thin or hard setules	with basal denticles	with denticles or hard setules in one species	with denticles or thin setules in three species	with thin setules	with thin setules
P1 – long anterior seta on endite 1	absent	absent	absent	absent	absent	absent	absent	absent	present
P2 – exopod seta	very short	short or moderated length	absent	absent	short	moderated length, rudimentary in two species	present in one species	absent	absent
P3 – number of setae on the exopod	six	seven	six	six	six	six	six	six	six
P4 – Number of flaming-torch	three	three	three	three	three	three	generally three, two in one species	two	three
**Male characters**									
A1 – lateral aesthetascs	six	two	two	two-six	two	absent	absent	one	unknown
PA – dorsal and ventral margins	almost parallel	parallel or narrowing	narrowing	narrowing	narrowing	almost parallel	almost parallel	almost parallel	unknown
PA – basal spine length	up 0.4 of postabdominal claw	absent or 0.5 of postabdominal claw	0.2–0.5 of postabdominal claw	0.1–0.3 of postabdominal claw	up 0.5 of postabdominal claw	0.2–0.5 of postabdominal claw	0.1–0.3 of postabdominal claw	0.1 of postabdominal claw	unknown
PA – position of gonopores	above of projection for claw insertion	above of projection for claw insertion	at base of postabdominal claw	above of projection for claw insertion or lateral	at base of postabdominal claw	above of projection for clauw insertion	above of projection for claw insertion	above of projection for claw insertion	unknown

#### Magnospina dentifera

Taxon classificationAnimaliaDiplostracaChydoridae

(Sars, 1901) comb. n.

Figures 1–7 , 8–15 , 16–26 , 27–33

Alona dentifera (Sars, 1901): Sinev et al. 2004: 101, 103–104, figures 1–39; Güntzel et al. 2010: 95, table 1; Sousa and Elmoor-Loureiro 2012: 356, table 2; Debastiani-Júnior et al. 2015: 24, table 2.
Alona broaensis : Matsumura-Tundisi and Smirnov 1984: 327–328, figures 15–21; Güntzel et al. 2010: 95, table 1; Debastiani-Júnior et al. 2015: 24, table 2.

##### Type locality.

“neighborhood of São Paulo”, State of São Paulo, Brazil.

##### Material type.

Lectotype: Parthenogenetic ♀, ZMOU F12341a, selected by D. Frey. Paralectotype: 5 parthenogenetic ♀♀, ZMOU F12341b; 1 parthenogenetic ♀ F12341c; 4 parthenogenetic ♀♀, I instar juvenile ♀, ZMOU F12386g; 2 instar II juvenile ♀ ZMOU F12386q; 2 partenogenetic ♀♀, instar II juvenile #, ZMOU, slide F9130; 6 parthenogenetic ♀♀ , ephippial ♀, ZMOU, slide F9131; 2 parthenogenetic ♀♀, ephippial ♀, ZMOU, slide F9131.

##### Material examined.

Nine parthenogenetic females and one adult male from Henrique pond, Brasília National Park, Distrito Federal, Brazil (15°41'18"S; 47°56'26.10"W), material collected by Grupo de Estudos de Ecossistemas Aquáticos (GEEA) in ix.2009 (FDRS048). One parthenogenetic female from Henrique pond, Brasília National Park, Distrito Federal, Brazil (15°41'16.5"S; 47°56'22.2"W), material collected by Lourdes M. A. Elmoor-Loureiro on 27.v.2002 (FDRS049). One parthenogenetic female from Cabocla II pond, Campo de Instrução de Formosa, Goiás, Brazil (15°48'21"S; 47°17'09.20"W), material collected by Grupo de Estudos de Ecossistemas Aquáticos (GEEA) on viii.2009 (FDRS050). Six adult parthenogenetic females and one juvenile from Baía da Célia, Fazenda Nhumirim (18°59'27.5"S, 56°39'41.0"W), Pantanal, Mato Grosso do Sul, Brazil, material collected on 07.ix.2000 by Valéria Barros. Four parthenogenetic females from Criminosa Pond (21°40'28.8"S, 57°53'28.5"W) identified as *Alona broaensis*, Porto Murtinho, Pantanal, Mato Grosso do Sul, Brazil, material collected on 19.i.2010, leg Adriana Maria Güntzel (FDRS054). Two parthenogenetic females from Coqueiral Pond, Paranapanema River, Angatuba, São Paulo, Brazil (23°29'22.64"S; 48°37'6.65"W). Material collected by Lourdes M. A. Elmoor-Loureiro on 30.v.2001 (CLLA063, 65-66). Two parthenogenetic females from Esquina, Middle Paraná River, Argentina (30°00.54'59"S; 59°32'51.93"W). Material collected by José Roberto Debastiani Júnior on 12.vi.2010 (FDRS052). Six parthenogenetic females from San Pedro, Lower Paraná River, Argentina (30°40'49"S; 59°18'48.80"W). Material collected by José Roberto Debastiani Júnior on 14.vi.2010 (FDRS053). Three parthenogenetic females from Pimenteira pond, Mata da Pimenteira State Park, Serra Talhada, Pernambuco, Brazil (7°53'48.96"S, 38°18'14.30"W). Material collected by Leidiane Pereira Diniz on 13.iv.2014 (FDRS407).

##### Differential diagnosis.

*Magnospina dentifera* comb. n. differs from *Magnospina siamensis* comb. n. because it has a rounded and wide rostrum and main and lateral head pores are absent in adult. Apical spines of the antenna about two times shorter than the apical segments. On the limbs, the main differences are: *Magnospina dentifera* comb. n. bears a rudimentary seta 1 on the IDL, setae 2-3 of IDL armed with at least seven denticles and the presence of one seta on the filter comb of limb V.

##### Diagnosis.

*Habitus* ovoid, without dorsal keel, not compressed laterally, length 0.32–0.48 mm; eye and ocellus of different sizes. *Head* with rostrum wide, rounded, not pointed; head shield wide, with broadly rounded posterior margin, distance between mandibular articulations higher than length of its posterior portion, main head pores absent in adults, two or three connected main head pores in juveniles (Figures 8–9); lateral head pores absent. *Labral keel* wide, large and naked, apex not elongated (Figure 10). *Carapace* ornamentation slightly punctuated or not evident; ventral margin of carapace with a distinctive rounded angle at 1/3 of length; valves armed with 40–53 setae internally inserted at the valve ventral margin and differentiated in three groups, setae from anterior group markedly longer than median and posterior groups (Figures 1–6); posteroventral corner armed with 1–4 large denticles, broad at their bases, protruding downwards, without setules between them (Figure 7). *Antennule* do not exceed the tip of the rostrum, nine apical aesthetascs of different lengths which do not exceed the length of the antennular body (Figure 11). *Antenna* with formula of antennal setae 003/113, spines 101/001; first segment of endopod and exopod elongated; weak setules or spicules on the segments; spine on the first segment of the endopod longer than second segment length; apical spines about two times shorter than the apical segments (Figure 12). *Postabdomen* narrowing distally, length about 1.3 times its height; preanal angle prominent; postanal margin about 1.5 times longer than the anal margin armed with 10–13 groups of denticles, 1–3 most distal denticle might be individualized; 8–10 lateral fascicles armed with weak setules (Figures 13–14). *Postabdominal claw* inserted on the projection of postabdomen, longer than anal margin; spinules on the ventral margin may be present; pecten of spinules on the internal and external face of the claw, base of claw armed with 1–5 long and strong spinules (Figures 14–15). *Basal spine* remarkably long, longer than 2/3 of postabdominal claw length, with spinules on the dorsal margin (Figures 14–15). *Limb I* with IDL (en 4) armed with one rudimentary seta (1) and two well-developed setae (2–3) which bears at least seven distinguishable denticles, basal denticles thick; seta (1) on endite 3 shorter than setae (a-c), setae (a-b) of different length, endite 3 with an element; endite 2 armed with three setae (d-f), element present; endite 1 with three setae (g-i) (Figures 16–19). *Limb II* without anterior soft setae; seta on the exopod short, slightly plumose; scrapers not specialized, but with some denticles, especially on scrapers 6–8; gnathobase armed with four elements, filter comb armed with seven setae, of which two are shorter than others (Figure 20). *Limb III* with six setae on the exopod, third and fourth setae long; third seta longer than the second seta; distal endite armed with three setae and one sensillum; gnathobase with three elements, filter comb with seven setae (Figures 21–22). *Limb IV* relatively short, six setae on the exopod; setae 1–2 of different lengths, flaming-torch setae on the distal endite not modified with weak setules; gnathobase armed with a setulated seta shorter than the length of endite itself, filter comb with five setae (Figures 23–26). *Limb V* relatively short, setae 3–4 of exopod similar in length; filter comb reduced with one short seta (Figures 25–26). *Limb VI* absent.

*Ephippial female*. Not studied.

*Adult male. Habitus* smaller than female (Figure 27). *Postabdomen* strongly narrowing distally. *Postabdominal claw* short and ticker than female (Figures 30–31). *Basal spine* about half-length of postabdominal claw, with tip forked (Figure 31). *Limb I* with two setae on the IDL (en4), setae armed with denticles; male seta with tip slightly curved; copulatory hook with one projection on the tip (Figures 32–33).

##### Description of adult male.

*Habitus* ovoid, smaller than that in female, length about 0.35 mm, maximum height in the middle of the body (Figure 27). *Head* with rostrum elongated, not blunt, main head pores absent (Figure 27). *Carapace* without ornamentations; ventral margin with a distinctive rounded angle at 1/2 of the margin length, margin armed with about 37 setae, posteroventral corner with two large denticles, broad at their bases, without setules between them (Figure 27). *Antennule* not exceeding the tip of rostrum, about 2.5 times as long as it is wide, with three rows of short setules on body antennular; eleven aesthetascs, two lateral and nine apical ones. Sensory seta and male seta not studied (Figures 27–28). *Antenna* as described for females, however, apical spines relatively longer (Figure 29). *Postabdomen* as long as in female, strongly narrowing distally. Anal margin shorter than postanal margin; 12 rows of thin setules on the anal and postanal margin; eight lateral fascicles with weak setules of which do not exceed postanal margin (Figure 30). *Postabdominal claw* smaller and more robust as comapared with female, base armed with long and strong spinule, pecten armed with strong spinules at the median portion of the claw (Figures 30–31). *Basal spine* long, about half-length of postabdominal claw, with a forked tip, ventral margin armed with spinules (Figure 31). *Limb I* with copulatory hook curved, U-shaped, projection at the tip present, copulatory brush seta shorter than male seta on IDL (en4), the latter armed with three setae; male setae thick with tip slightly curved; setae 2–3 armed with proximal denticles (as observed in female); ODL seta longer than IDL setae (Figures 32–33).

##### Distribution.

Neotropics, from Southern U.S.A to Argentina (Sinev et al. 2004).

#### Magnospina siamensis comb. n.

Taxon classificationAnimaliaDiplostracaChydoridae

Alona siamensis : Sinev and Sanoamuang 2007: 145, 147–148, figures 1–30; Van Damme and Sinev 2013: 226–228; Korovinchinsky 2013: 114, 119, tables 1–2.
Coronatella dentifera (Sars, 1901): Chartejee et al. 2013: 43.

##### Type locality.

Rice field at Ban Bayao Baghe Sub-district, Phannanichom District, Sakhonnakhon Province, Thailand, 01.09.2004.

##### Material type.

Holotype: parthenogenetic female, ZMMU, MI-73. Paratypes: 2 parthenogentic females, ZMMU, MI74.

##### Differential diagnosis.

*Magnospina siamensis* comb. n. differs from *Magnospina dentifera* comb. n. because it has a truncated rostrum, three connected main head pores, minute lateral head pores and a prominent sculpture on the carapace. Apical spines of the antenna are longer than the apical segments. On the limbs, the main differences are: IDL is armed with two setae (2-3), seta 2 with two thick basal denticles, seta 3 with one thick basal denticle, limb V without filter comb.

##### Diagnosis.

**Female.** According to the literature (Sinev and Sanoamuang 2007).

*Habitus* without dorsal keel, moderately compressed laterally, length 0.35–0.42 mm; eye and ocellus of subequal sizes. *Head* with rostrum wide, truncated; head shield ornamented with longitudinal lines, wide, posterior margin broadly rounded, distance between mandibular articulations higher than length of its posterior portion, three connected main head pores, lateral head pores minute. *Labral keel* wide, oval and naked, apex not elongated. *Carapace* covered with narrow longitudinal lines; ventral margin of carapace with a distinctive rounded angle at 1/3 of length; valves armed with 45 setae internally inserted at the ventral margin and differentiated in three groups, setae from anterior group markedly longer; posteroventral corner armed with 2–3 large denticles, broad at their bases, protruding downwards, without setules between them. *Antennule* do not exceed the tip of the rostrum, nine apical aesthetascs of different length which do not exceed the length of the antennular body. *Antenna* with formula of antennal setae 003/113, spines 101/001; first segment of endopod and exopod elongated; weak setules or spicules on the segments; spine on the first segment of the endopod longer than the second segment; apical spines longer than the apical segments. *Postabdomen* narrowing distally, length about 2.5 it is height; preanal angle prominent; postanal margin about 1.7–1.8 times longer than the anal margin armed with 3–4 single most distal denticles and 5 cluster of denticles; About 10 lateral fascicles with weak setules. *Postabdominal claw* inserted on the projection of postabdomen, longer than anal margin; base of claw armed with 1–2 long and strong spinules. *Basal spine* remarkably long, longer than 2/3 of postabdominal claw length, without spinules on the dorsal margin. *Limb I* with two thick setae (2–3) on the IDL (en 4), seta (2) with two thick basal denticles, seta (3) with one thick basal denticle; setae (1, a-c) on the endite 3 of subequal length, endite 3 without element; endite 2 armed with three setae (d-f), without element; endite 1 with three setae (g-i). *Limb II* without anterior soft setae; seta on the exopod short, not plumose; scrapers not specialized, but with some denticles, especially on scrapers 6–8; gnathobase armed with four elements, filter comb armed with seven setae, of which two are shorter than the others. *Limb III* with six setae on the exopod, third and fourth setae long; third seta slightly shorter than the second seta; distal endite armed with three setae and one sensillum; gnathobase with three elements, filter comb with seven setae. *Limb IV* relatively short, six setae on the exopod; setae 1–2 of similar lengths; flaming-torch setae on the distal endite not modified, setules on the first flaming-torch relatively longer that one observed in setae 2–3; gnathobase armed with a setulated seta shorter than length of endite itself, filter comb with five setae. *Limb V* with setae 3–4 of exopod similar in lengths; filter comb absent. *Limb VI* absent.

**Ephippial female and male.** Unknown.

##### Distribution.

Malysia, Thailand (Sinev 2007; Van Damme and Sinev 2013; Korovinchinsky 2013) and probably India (Chartejee et al. 2013).

## Discussion

### Morphological analyses

In the redescription of *Alona dentifera*, Sinev et al. (2004) suggested that specific morphological traits observed in this species were not enough to create a new genus, however, the description of *Alona siamensis* (Sinev and Sanoamuang 2007) showed a new perspective about *dentifera*-group. Thus, separation of *Magnospina* gen. n. is mainly supported by such characters as: (1) basal spines longer than 2/3 of postabdominal claw, (2) presence of 1-4 large denticles, broad at their bases, protruding downwards, without setules between them. Other specific morphological traits also are observed in *Magnospina* gen. n.: presence of a distinctive rounded angle at 1/3 of the length of ventral margin of carapace, setae on the valves differentiated in three groups with the anterior group markedly longer than median and posterior groups, prominent preanal angle at postabdomen, setae 2–3 of IDL armed with basal denticles, six setae on limb III, and absent limb VI. The presence of six setae on exopod of limb III and absence of limb VI may be considered as simplesiomorphies of the clade *Magnospina dentifera*/ *Magnospina siamensis*, and ancestral state for the *Coronatella*-branch.

The morphology of head shield, main head pores and of some structures of the limbs are different between *Magnospina dentifera* comb. n. and *Magnospina siamensis* comb. n.; however, analogous variation in these structures was already observed in *Euryalona* (Rajapaksa and Fernando 1987). Species-groups of *Alona* sensu lato, such as the *costata*-group (Sinev 1999b, 2001a, 2008, Van Damme and Eggermont 2011, Van Damme et al. 2011b), *verrucosa*-group (Van Damme et al. 2011a, Sinev and Kotov 2012), *rectangula*-group (Van Damme and Dumont 2008b, Sousa et al. 2015a), and *pulchella*-group (Sinev 2001b, c, 2009, Sinev and Silva-Briano 2012, Van Damme et al. 2013, Sousa et al. 2015b) also have differences in structures on head, postabdomen, and limbs.

Recently, Sinev (2014, 2015b) reviewed the morphology of *Camptocercus* Baird, 1843 species and showed significant differences in structures on the limbs among different species of this genus. In the same way, *Celsinotum* also has many differences in the morphology of head shield, postabdomen and limbs (Frey 1991, Sinev and Elmoor-Loureiro 2010, Sinev and Kotov 2012). This endorses our conclusion that the differences between *Magnospina dentifera* comb. n. and *Magnospina siamensis* comb. n. should be considered at a specific level in the *dentifera*-group (also suggested by Sinev and Sanoamuang 2007). For Van Damme and Sinev (2013), this small lineage may represent an ancient vicariant divergence, presenting currently an Amphi-Pacific distribution, i.e. keeping in mind an antiquity of the cladoceran taxa of different ranks (Frey 1987; Kotov and Taylor 2011). Aforementioned differences in the morphology between *Magnospina dentifera* comb. n. and *Magnospina siamensis* comb. n. may be the result of adaptations to different environmental pressure on a micro-scale.

The trend in morphological radiation in the clade *Magnospina* gen. n. concerns the external morphology but not to features of the trunk limbs (such as in the *pulchella*-group). It has been observed that a wide rostrum and the maintenance of primitive ovoid body shape, shared with other species-groups, possibly result from convergence or parallelism (Sinev et al. 2005, Van Damme and Dumont 2008b, Sinev et al. 2009, Van Damme and Sinev 2011). Regarding the limbs, an exception to the aforementioned trend seems be the armature of the IDL setae, which is more specialized in *Magnospina siamensis* comb. n. when compared to *Magnospina dentifera* comb. n. (which has the armature of IDL setae similar to genus *Coronatella*). A similar trend was observed in species of *Anthalona* whose evolution of IDL setae are related to feeding strategies (Van Damme et al. 2011a). Thus, distinct evolutionary pressure on the food handling should be considered to explain differences observed on the IDL setae of *Magnospina* gen. n. species.

The morphology of the postabdomen is the most evident trait of *Magnospina* gen. n. in contrast to *Leberis*, *Coronatella*, *Anthalona*, *Karualona*, *Extremalona*, *Bergamina*, *Celsinotum*, *Ovalona* or *Alona* senso stricto; however, this morphological feature does not show a clear relationship with habitat and/or evolutionary history. Generally, specialized species have their morphology linked to habitat conditions (Van Damme et al. 2003, Kotov 2000a, b, Kotov 2006, Van Damme et al. 2009, Kotov et al. 2010, Van Damme and Sinev 2011, Van Damme et al. 2011a, Sinev 2014), but, apparently, this is not case of the two species from the *dentifera*-group, because they may occur in different kinds of habitats (Sinev and Sanoamuang 2007, Güntzel et al. 2010, Sousa et al. 2012, Kotov et al. 2013, Van Damme and Sinev 2013).

Some studies observed that the male’s morphology is very important in making any inference about the relationship between closer species or between species groups in Aloninae (Sinev 1999a, 2013) as well as other cladoceran groups (Goulden 1968; Belyaeva and Taylor 2009; Kotov et al. 2009). Indeed, the morphology of the *Magnospina dentifera* comb. n. male indicates more affinities with *Leberis* than with any genus from the *Coronatella*-branch. For instance, the general shape of postabdomen and antennules is similar to that described for adult males of *Leberis davidi* (Richard, 1895) (Sinev et al. 2005) and *Leberis colombiensis* Kotov & Fuentes-Reines 2015 (Kotov and Fuentes-Reines 2015). However, there are clear differences between *Leberis* and *Magnospina* gen. n.: the presence of denticles on posteroventral corner of valves, shape of postabdominal claw, length of the basal spine, and armature of IDL setae (for *Magnospina dentifera* comb. n., Figures 30–32).

When evaluating the morphology of species of the *elegans*-group from Palearctic zone, Sinev et al. (2009) highlighted the morphological traits that support that this group in *Coronatella*-branch, as well as its presumed genus-level. Thus, the main difference between females from *Magnospina* gen. n. and species of the *elegans*-group are related to the external morphology (shape of the body, rostrum, postabdomen and presence of denticles on the posteroventral corner of the carapace in *Magnospina* gen. n.); differences on the limbs are observed in the armature of IDL setae and the length of the seta 3 on the exopod of limb III. Males from the *elegans*-group share with *Magnospina* gen. n. the presence of two lateral aesthetascs on the antennules (Figure 28), which is considered the main synapomorphy of clade *Ovalona*/*elegans*-group/*Leberis* (Sinev 2015a; Neretina and Sinev 2016). This confirms the phylogenetic position of *Magnospina* gen. n., which is closely related to *Leberis*.

The male of *Ovalona* genus also has two lateral aesthetascs on the antennules, but differs from *Magnospina* gen. n. because it has straight dorsal and ventral postabdominal margins, gonopores opening above projection to insertion of postabdominal claw, and setae 2–3 of IDL armed with setules. According to Sinev (2015a) and Neretina and Sinev (2016), *Celsinotum* is closer to *Leberis* and *Ovalona*, and thus, its close relationship with *Magnospina* gen. n. could be inferred. *Celsinotum* females differ quantitatively from *Magnospina* gen. n. in external and limb structures (see Frey 1991, Sinev and Elmoor-Loureiro 2010, Sinev and Kotov 2012). The males of *Celsinotum* differ from *Magnospina* gen. n. in the shape of postabdomen, length of the basal spines on the postabdominal claw, presence of two-six lateral aesthetascs on antennules and setae 2–3 of IDL armed with setules (see Frey 1991, Sinev and Kotov 2012).

Differently from *Magnospina* gen. n., males of the *Coronatella* genus have dorsal and ventral margins of the postabomen almost straight and lateral aesthetascs on antennules absent (Van Damme and Dumont 2008a, Sousa et al. 2015a). *Anthalona* males have a short basal spine, well-developed setules of the lateral fascicles on the postabdomen and lateral aesthetascs on antennules absent (Van Damme et al. 2011a, Sinev and Kotov 2012). *Karualona* males has postabdomen very similar to the one observed in *Anthalona*, with well-developed lateral fascicles on the postabdomen and a very short basal spine on the postabdominal claw. However, antennules of *Karualona* males bear one lateral and ten apical aesthetascs (see Alonso and Petrus 1989). Besides short postabdomen, the male of *Extremalona* has six lateral aesthetascs on antennules and well developed seta 1 of IDL and setae 2–3 armed with setules (Sinev and Shiel 2012). Differences between *Magnospina* gen. n. male and *Bergamina* cannot be stated because the male is not known, so far.

### Notes on *Alona broaensis* Matsumura-Tundisi & Smirnov, 1984

*Alona broaensis* species was described from Broa Reservoir, São Paulo, Brazil (Matsumura-Tundisi and Smirnov 1984) and it has not often been found in fauna studies conducted in many regions (including type region). The absence of some information on the morphology, including details from trunk limbs, led Van Damme et al. (2010) to list this species as a junior synonym of *Magnospina dentifera* comb. n.. Indeed, the morphological variation observed between *Magnospina dentifera* comb. n. populations studied here and by Rey and Vasquez (1986) for number of denticles on the posteroventral corner of carapace (Figure 8), morphology of the postabdominal claw, basal spine, and rostrum, include the features signed as diagnostic for *Alona broaensis* (see Matsumura-Tundisi and Smirnov 1984). They are like those observed in description of *Alona broaensis* (see Matsumura-Tundisi and Smirnov 1984). We analyzed one population identified as *Alona broaensis* from the Pantanal, Brazil, and morphological traits distinct from *Magnospina dentifera* comb. n. were not observed. In the other words, there are not morphological traits that support the validity of *Alona broaensis*. We agree with the suggestion of Van Damme et al. (2010), and *Alona broaensis* is here considered as a junior synonym of *Magnospina dentifera* comb. n..

## Conclusions

*Magnospina* gen. n. is one more genus derived from *Alona* sensu lato and belongs to the *Coronatella*-branch, being close to *Leberis*, as suggested by the phylogenetic analysis based on molecular data. The synapomorphies of the *Magnospina* gen. n. are: (1) basal spines longer than 2/3 of postabdominal claw, (2) presence of 1-4 large denticles, broad at their bases, protruding downwards, without setules between them. *Magnospina* gen. n. also has a wide rostrum, prominent preanal angle at postabdomen, setae 2-3 of IDL armed with basal denticles, six setae on limb III and limb VI absent. In addition to the female morphology presenting consistent differences when compared to other genera from the *Coronatella*-branch, the male features also support the creation of this new genus that includes *Magnospina dentifera* comb. n. and *Magnospina siamensis* comb. n.

## Supplementary Material

XML Treatment for Magnospina

XML Treatment for Magnospina dentifera

XML Treatment for Magnospina siamensis
